# Impact of Program–Erase Operation Intervals at Different Temperatures on 3D Charge-Trapping Triple-Level-Cell NAND Flash Memory Reliability

**DOI:** 10.3390/mi15091060

**Published:** 2024-08-23

**Authors:** Xuesong Zheng, Yifan Wu, Haitao Dong, Yizhi Liu, Pengpeng Sang, Liyi Xiao, Xuepeng Zhan

**Affiliations:** 1 School of Astronautics, Harbin Institute of Technology, Harbin 150001, China; zhengxuesongkx@163.com (X.Z.); xiaoly@hit.edu.cn (L.X.); 2 China Aerospace Components Engineering Center, Beijing 100094, China; 3 School of Information Science and Engineering, Shandong University, Qingdao 266237, China; 202332702@mail.sdu.edu.cn (Y.W.); dhtzzjbyl@126.com (H.D.); lyz2308237138@163.com (Y.L.); ppsang@sdu.edu.cn (P.S.)

**Keywords:** flash reliability, 3D CT NAND, operation interval, high temperature, TLC

## Abstract

Three-dimensional charge-trapping (CT) NAND flash memory has attracted extensive attention owing to its unique merits, including huge storage capacities, large memory densities, and low bit cost. The reliability property is becoming an important factor for NAND flash memory with multi-level-cell (MLC) modes like triple-level-cell (TLC) or quad-level-cell (QLC), which is seriously affected by the intervals between program (P) and erase (E) operations during P/E cycles. In this work, the impacts of the intervals between P&E cycling under different temperatures and P/E cycles were systematically characterized. The results are further analyzed in terms of program disturb (PD), read disturb (RD), and data retention (DR). It was found that fail bit counts (FBCs) during the high temperature (HT) PD process are much smaller than those of the room temperature (RT) PD process. Moreover, upshift error and downshift error dominate the HT PD and RT PD processes, respectively. To improve the memory reliability of 3D CT TLC NAND, different intervals between P&E operations should be adopted considering the operating temperatures. These results could provide potential insights to optimize the lifetime of NAND flash-based memory systems.

## 1. Introduction

In the era of big data and information explosion, 3D NAND flash memory is widely used in various applications benefiting from unique advantages including ultra-high storage density, storage capacities, and low product cost [1,2,3,4]. Three-dimensional NAND flash memory is becoming a mainstream storage medium, which is the core of a solid state disk (SSD) and provides great potential to replace a hard disk drive (HDD). Extensive efforts have been devoted to improving NAND flash memory by exploring the fabricating process, novel materials, and device architectures [5,6,7]. During the operation process, repeated Program and Erase (P/E) operations bring about unavoidable degradations in the vertical tunneling layer (TNL) and charge trapping layer (CTL), leading to worse reliability and performance [8,9,10]. J. K. Park et al. focus on the cell operation algorithm and scheme to improve the reliability of NAND cells [11]. For large-scale and highly reliable memory devices and systems, in-depth investigations on the reliability characteristics and the potential failure mechanism are of great importance.

Program disturbance (PD), also as the repeated P/E operation, originates from the unselected cells in 3D NAND flash sharing a common word line (WL) for the selected and unselected cells [12,13,14,15]. The PD phenomenon becomes exacerbated as the stacking storage layer increases, which is related to the leakage current through the vertical tunneling layer (TNL) under a low electric field and lateral charge migration (LCM) from the adjacent flash cells [16,17]. Several methods have been proposed to reduce PD-induced errors, including pre-charge operation of the unselected cell, self-boosting effect, and optimized programming scheme [18,19]. However, the impacts of the intervals between P&E operations under different operating temperatures on the error distributions and error mode of 3D NAND flash are rarely reported.

In this paper, the impacts of the intervals between P&E operations on 3D TLC NAND flash are investigated under room temperature and high temperature, at fresh states and up to 10 K P/E cycling states. With a constant operation period of 0.1 s, two groups of Ters (the interval between Erase and Program) and Tpgm (the interval between Program and Erase) were adopted, which are 0.01 s and 0.09 s, and 0.09 s and 0.01 s, respectively. The impacts of threshold voltage (Vth) shifts were analyzed by focusing on the error bit states, the operating temperature, and the P/E cycles. We found that the intervals between P&E operations, as well as the operating temperatures, show obvious impacts on the error modes and total error bits, which indicates that longer Ters are preferred to reduce the error bits for fresh states up to 10 K P/E cycle state at room temperature.

## 2. Methods

An FPGA-based raw NAND chip tester was adopted to characterize the Raw 3D TLC NAND chips, which has eight parallel sockets and high-speed PCIE interfaces. The tester can support a maximum data transfer of ~200 MHz and a wide temperature range for operations, from 25 °C (room temperature, RT) to 85 °C (high temperature, HT). Customized software was used to perform the data program, data erase, and data read operations for detailed analysis. The tested Raw 3D TLC NAND chip was 128 G CT type flash memory with 64 stacking storage layers and 5912 valid blocks, which have 768 logical pages and store 18,336 bytes per page. The interval between Erase and the next Program operation is defined as Ters and the interval between Program and the next Erase operation was defined as Tpgm. Two groups of operation intervals were used corresponding to Ters and Tpgm of 0.01 s and 0.09 s, and 0.09 s and 0.01 s, respectively. The program disturb (PD) was evaluated by performing the repeated P/E to 2000 (2 K) cycles with a constant operation interval of 0.1 s and a read dummy operation per 200 cycles. The PD characterization was carried out at two different temperatures (25 °C and 85 °C) and six different P/E states (fresh, 2 K P/E cycles, 4 K P/E cycles, 6 K P/E cycles, 8 K P/E cycles and 10 K P/E cycles). After the Erase operation, a fixed set of pseudo-random data was used for the Program operation. The fail bit counts (FBCs) and the raw bit error rate (RBER) were analyzed by comparing the written and readout data. The read disturb (RD) and data retention (DR) experiments were performed after PD processes. The read dummy operations were conducted at 1st, 100th, 200th, 500th, 1000th (1 K), 2000th (2 K), 5000th (5 K), 8000th (8 K), and 10,000th (10 K) during the RD process. The read dummy operations were conducted at 0 h, 1 h, 2 h, 4 h, 6 h, 12 h, 18 h, and 24 h during DR process. In this work, the PD process was carried out under RT and HT conditions, while the following RD and DR measurements were performed at RT.

**Figure 1:**
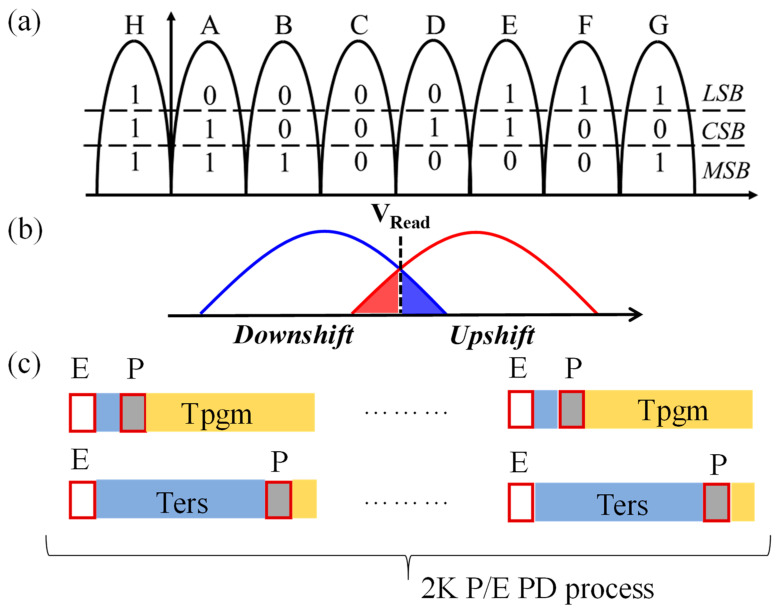
Schematic images of (**a**) Vth distribution (H to G) in TLC NAND flash, and (**b**) the upshift (blue region) and downshift (red region) error with a given read voltage (Vread). (**c**) Schematic of Program and Erase operations with the interval defined as Tpgm and Ters. The white squares and grey squares represent the Erase (E) and Program (P) operations.

Figure 1c shows the experimental process, which contains 2 K P/E cycles (PD) with different operation intervals. During the PD process, the repeated Erase operation was performed followed by the repeated Program operation with the fixed random data. Typically, incremental step pulse programming (ISPP) and incremental step pulse erase (ISPE) are adopted, which contains multiple pulses with verify pulses to check the memory states. When P&E operations are completed, there are periods of waiting time corresponding to Ters and Tpgm, respectively. During the RD process, repeated data Read operations ranging from 1 cycle to 10,000 (10 K) cycles were performed after the PD process. During the DR process, repeated data Read operations were performed immediately after the PD process and remaining up to 24 h. For the data analysis process, the readout data were recorded and downloaded to a text file and then compared with the fixed random data. In the 3D CT TLC NAND flash chip, the stored data were divided into eight states based on the threshold voltage (Vth) of the storage cell corresponding to H, A, B, C, D, E, F, and G. As shown in Figure 1, there is a wide margin between adjacent memory states under ideal conditions. The adjacent memory states in practice overlap to a certain extent, leading to the error bits. The total error bits come from two parts defined as downshift errors and upshift errors, corresponding to the red region and blue region in Figure 1b.

## 3. Results and Discussion

**Figure 2:**
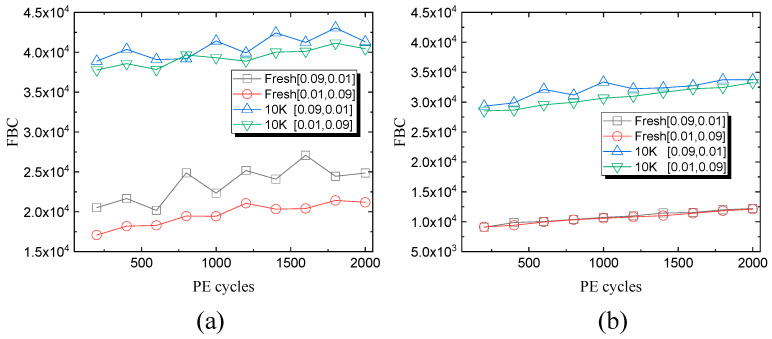
FBCs during 2 K P/E cycles with different Tpgm and Ters under fresh and 10 K P/E cycling states at (**a**) room temperature (25 °C) and (**b**) high temperature (85 °C).

Figure 2 shows the measured FBC under different Ters and Tpgm at fresh blocks and 10 K P/E cycling blocks. It vividly shows that the FBCs of 10 K P/E cycling blocks were significantly larger than those of fresh blocks under 25 °C and 85 °C conditions. During the room-temperature (RT) PD process, the longer Tpgm shows smaller FBCs both at fresh and 10 K P/E cycling states. This can be explained through the fact that a longer Tpgm shows a higher possibility for the stored charge loss through the LCM, which compensates for the slight injected charges during the PD process. Similar results were observed during the high-temperature (HT) PD process. With the first FBCs extracted at room temperature before the PD process as a reference, the FBCs showed a significant decrease at small PE cycle numbers. The FBCs increased slowly with increasing PE cycles, which can be explained through the fact that the flash memory behaves more reliably at a high temperature. Note that different operation intervals show little difference at the fresh state.

**Figure 3:**
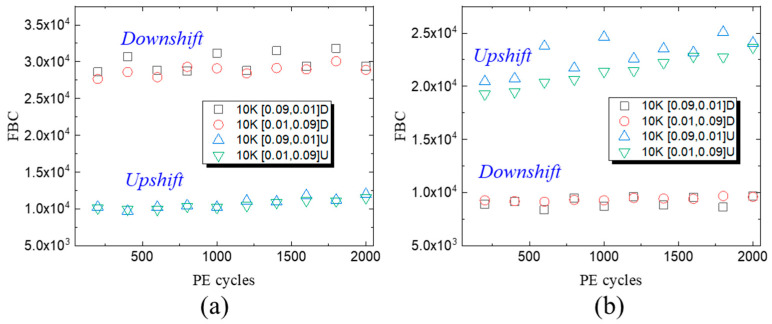
The corresponding downshift and upshift error at (**a**) 25 °C and (**b**) 85 °C during 2 K P/E cycles with different Tpgm and Ters.

Considering the overlap between the memory states in 3D NAND flash with the triple-level-cell (TLC) mode, the errors can be divided into Vth-downshift errors and Vth-upshift errors, as shown in Figure 1. After 10 K P/E cycles, the downshift and upshift errors are displayed in Figure 3 with different intervals between P&E operations and operating temperatures. For the RT PD process, the downshift errors overwhelm the upshift errors, while the opposite trend was observed at the HT PD process. A roughly linear relationship between the upshift errors and P/E cycles can be observed in Figure 3b at a high temperature. Depending on the stored charge levels, Vth-downshift errors and Vth-upshift errors roughly correspond to the charge loss and charge accrual process. Normally, charge loss can happen through lateral migration between adjacent cells and vertical migration between the blocking layer and tunneling layer. The upshift errors are related to the vertical charge injection from the tunneling layer, the lateral charge migration within the charge-trapping layer, and the set of Read voltage [20,21,22,23,24,25,26]. As P/E cycling increases, more shallow traps are generated under P/E stress, and the charge accrual in low Vth states becomes one dominant factor for worse FBCs [27,28]. The increasing upshift errors at different P/E cycles are related to the generated shallow traps and degradation of the tunneling layer.

In order to further reveal the mechanism of upshift errors, shown in Table 1, the upshift errors of each state were summarized with different temperatures and operation intervals. It is obvious that the low Vth state errors are the dominant factor for upshift errors, which correspond to H-to-A state errors at room temperature and A-to-B state errors at a high temperature for both the operation intervals of 0.09 s and 0.01 s, and 0.09 s and 0.01 s.

**Figure 4:**
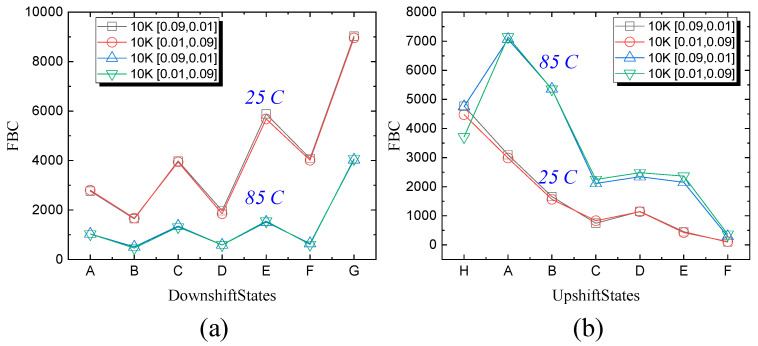
The FBC summary of different states in (**a**) downshift errors and (**b**) upshift errors after 10 K P/E cycles with different Tpgm and Ters at 25 °C and 85 °C.

Furthermore, the error of each state (H, “111” to G, “101”) of the TLC modes are displayed in Figure 4 with different operation intervals and operating temperatures. Only the results of 10 K P/E cycling blocks were investigated to reveal the differences. The G-state and E-state downshift errors were much larger than those of the others states under RT and HT conditions, which are more likely to lose charges owing to the higher Vth states. For upshift errors, the A-state and B-state errors are dominant under HT conditions, while the H-state and A-state errors are larger than others under RT conditions. The results indicate that the operating temperatures are more important on the error modes than operation intervals.

**Figure 5:**
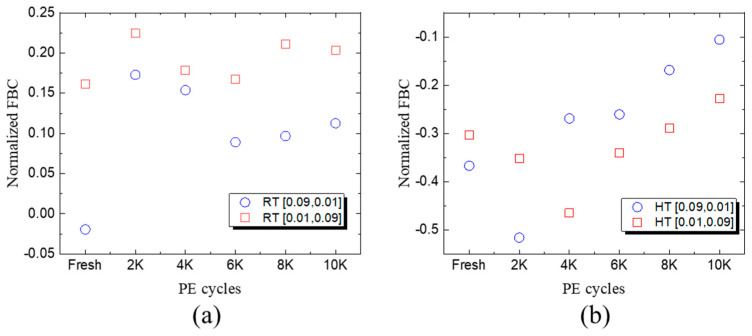
The normalized FBC under different P/E cycles (fresh to 10 K) with different Tpgm and Ters at (**a**) 25 °C and (**b**) 85 °C.

**Figure 6:**
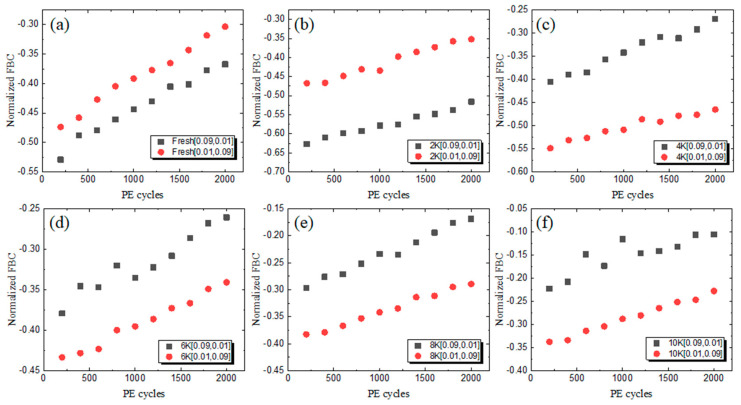
The normalized FBC versus P/E cycles with different P/E cycling states at 85 °C and different operation intervals: (**a**) fresh state; (**b**) 2 K P/E cycling; (**c**) 4 K P/E cycling; (**d**) 6 K P/E cycling; (**e**) 8 K P/E cycling; and (**f**) 10 K P/E cycling.

Aiming to reveal the impacts of different operation intervals and operating temperatures, the normalized FBCs are shown in Figure 5 from fresh states up to 10 K P/E cycling states (2 K P/E, 4 K P/E, 6 K P/E, 8 K P/E, and 10 K P/E). After the RT PD process, a shorter Tpgm showed smaller normalized FBCs compared to that of a longer Tpgm from fresh state to 10 K P/E cycling states, which corresponds to less charged electron loss during Tpgm. When it comes to the HT PD process, the raw FBCs show little differences with different operation intervals (0.09 s and 0.01 s, and 0.01 s and 0.09 s) from fresh states to 8 K P/E cycling states. In order to reveal the differences, the normalized FBCs were extracted with a reference FBC (at room temperature) before the PD process. Detailed information is displayed in Figure 6, in which the normalized FBCs are below zero owing to the smaller FBCs at a high temperature. It shows an obvious change between the 2 K P/E cycling state and 4 K P/E cycling state, which corresponds to the results shown in Figure 5b. From the fresh state to 2 K P/E cycling states, shorter Tpgm operation intervals are also preferred, which is approximately equal to the lifetime of 3D TLC NAND flash memory. While after the typical lifetime (4 K P/E cycles to 10 K P/E cycles), it is interesting that a longer Tpgm shows smaller normalized FBCs. The stored charges (after Program operation) are lost during Tpgm, and a longer Tpgm implies a higher opportunity to lose the stored charges. At the HT PD process where the dominant factor is the upshift error, it was supposed that the FBCs could be compensated to a certain extent under a longer Tpgm, during which more stored charges can be lost through lateral charge diffusion.

**Figure 7:**
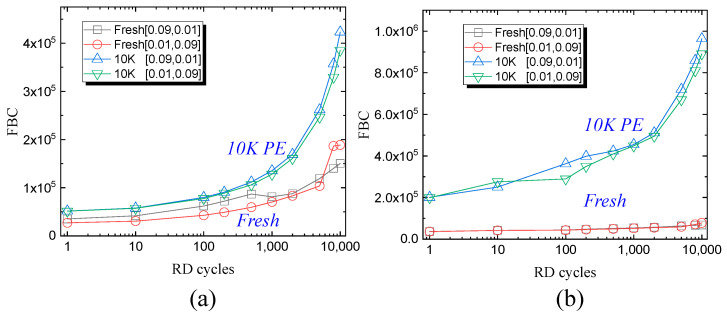
The 10 K read disturb error of fresh block and 10 K P/E cycling blocks with different Tpgm and Ters at (**a**) 25 °C and (**b**) 85 °C.

After different PD processes, the RD results are shown in Figure 7 at the fresh state and 10 K P/E cycling states. At up to 10 K read cycles at room temperature, the FBCs increase rapidly for fresh blocks and 10 K P/E cycling blocks after the RT PD process, which indicates the stored charges gradually lost during the RD process. Similar results were observed for the HT PD process shown in Figure 7b. Moreover, after the HT PD process, the FBCs at 10 K P/E cycling states were much larger than those of the RT PD process, which can be explained through the fact that more stored charges corresponding to dominant upshift errors can be lost during the RD process. The FBCs of the fresh block remain constant during 10 K RD cycles after the HT PD process, which is much smaller than those after the RT PD process. These results are in accordance with smaller FBCs obtained under the HT PD process.

**Figure 8:**
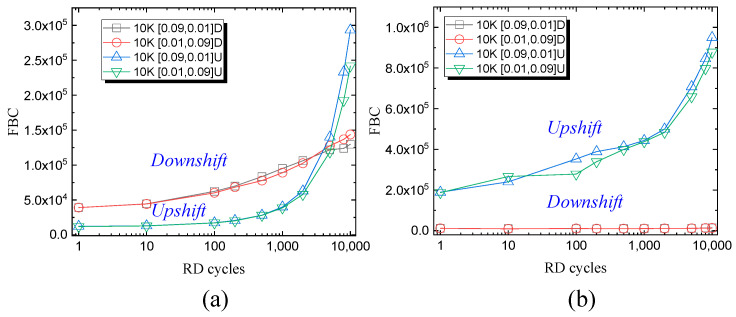
The downshift and upshift error during the 10 K read disturb after 10 K P/E cycles with different Tpgm and Ters at (**a**) 25 °C and (**b**) 85 °C.

To further reveal the underlying mechanism during 10 K read cycles, the downshift and upshift errors at 10 K P/E cycling with different operation intervals and operating temperatures are displayed in Figure 8. After the HT PD process, the upshift errors increase rapidly while the downshift errors remain unchanged up to 10 K RD cycles. On the contrary, the upshift errors increase rapidly and overwhelm the downshift errors after 5 K RD cycles after the RT PD process. During the RD process, the fundamental reason for the upshift errors is vertical charge injection through the tunneling layer. Along with P/E cycling, lateral charge migration will be another factor for charge accrual. The downshift errors are related to the loss of electrons stored in the charge trap layer through either lateral migration or vertical migration. As shown in Figure 7, the FBCs of the 10 K P/E cycling block after the HT PD process are much larger than those after the RT PD process. Depending on the charge loss and charge accrual degree, upshift errors or downshift errors are the dominant factor for error bits. The loss of electrons will be worsened and cause more downshift errors at high temperatures. In floating-gate (FG)-type NAND flash memory, TNL degradation dominates the downshift errors; however, in CT-type NAND flash memory, the charges are stored in charge-trapping centers, which are stable even with TNL degradation. Instead, the generated shallow traps in the CT layer turns to be the dominant factor for charge-loss. In previous work, it has been evidenced that P/E stress will generate shallow traps that contribute to the degradation of disturbance and retention [27,28].

**Figure 9:**
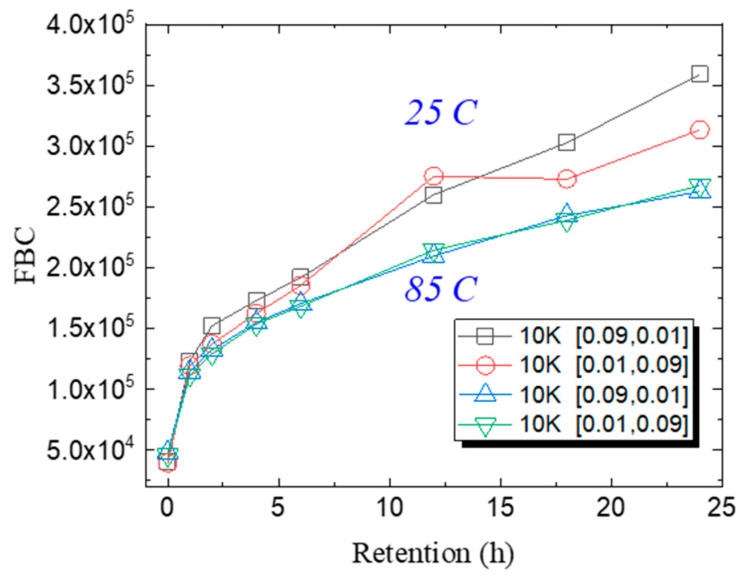
The 24 h retention results after 10 K P/E cycles with different Tpgm and Ters at 25 °C and 85 °C.

Figure 9 shows the 24 h DR results of the 10 K P/E cycling block with different operation intervals and operating temperatures. It shows that the FBCs after the HT PD process were similar for different operation intervals in which charge loss is dominant in the DR process. The FBCs of the RT PD process are much larger than those of the HT PD process. For the RT PD process, it was observed that a longer Tgpm (red circles) shows smaller FBCs than that of longer Ters (black squares). This indicates that stored charges are re-distributed and loss during a longer Tgpm through the LCM or vertical charge migration resulting in a smaller FBCs in DR.

## 4. Conclusions

In summary, this study investigated the impacts of operation intervals on 3D TLC NAND flash. The experimental measurements were conducted with a constant operation period of 0.1 s at 25 °C and 85 °C, at fresh states and up to 10 K P/E cycling states. It was found that upshift errors dominate during the high-temperature PD process, while downshift errors dominate during the room-temperature PD process. The total error bits of the HT PD process were much smaller than those of the RT PD process, of which the trends were similar for fresh blocks and up to 10 K P/E cycling blocks. Moreover, less degradation can be obtained using a longer Tpgm and higher operating temperature, which may be related to an upshift error originating from tunneling-induced leakage current. Therefore, different optimizing strategies should be adopted considering the operating temperature and imposed P/E cycling for 3D CT NAND flash.

## Figures and Tables

**Table 1:** The summary of upshift errors under 25 °C and 85 °C temperature conditions.

States	RT1 FBC	HT1 FBC	RT2 FBC	HT2 FBC
H To A	4771	4753	4477	3709
A To B	3097	7069	2984	7145
B To C	1659	5359	1563	5352
C To D	746	2111	829	2246
D To E	1149	2344	1137	2480
E To F	449	2148	420	2364
F To G	96	289	112	363

RT and HT stand for room temperature and high temperature, respectively, and 1 and 2 stand for Tpgm of 0.09 s and 0.01 s, respectively. The maximum values are highlighted in blue (RT) and red (HT).

## Data Availability

Data are contained within the article.
